# Success and failures in MRSA infection control during the COVID-19 pandemic

**DOI:** 10.1186/s13756-022-01158-z

**Published:** 2022-09-25

**Authors:** Kevin T. Kavanagh, Lindsay E. Cormier

**Affiliations:** 1 Health Watch USA, Somerset, KY 42503 USA; 2 Health Watch USA, Eastern Kentucky University, Lexington, KY 40509 USA

**Keywords:** MRSA, Veterans Health Administration, VHA, Pandemic, SARS-CoV-2, healthcare associated infections, NHSN, SIR, Staffing, ADI, Active surveillance, Contact precautions

## Abstract

Private sector facilities in the United States have experienced a resurgence of Methicillin-resistant *Staphylococcus aureus* (MRSA) hospital-onset infections during the COVID-19 pandemic, which eliminated all gains that were achieved over the last decade. The third quarter of 2021, the Standardized Infection Ratio for hospital onset MRSA bloodstream infections was 1.17, well above the baseline value of 1.0. In contrast, the Veterans Health Administration (VHA) has been able to maintain its mitigation efforts and low rates of MRSA hospital-onset infections through the second quarter of fiscal year 2022 (Mar. 31, 2022), the most recent available data. The difference may be explained not only by the VHA’s use of uniform mitigating policies which rely on active surveillance and contact precautions, but also on the VAH’s ability to maintain adequate staffing during the pandemic. Future research into MRSA mitigation is warranted and this data supports the need for healthcare system transformation.

Private sector facilities have long struggled to control Methicillin-resistant *Staphylococcus aureus* (MRSA) infections, a situation made worse by the COVID-19 pandemic. Reporting of MRSA hospital onset bacteremia became available through the National Healthcare Safety Network (NHSN) in 2009. A 2010–2011 baseline was adopted for the reporting of a Standardized Infection Ratio (SIR). The SIR is a risk-adjusted rate of MRSA bacteremia per patient bed days. The SIR was set to a baseline value of 1.0. Facilities having an SIR lower than 1.0 have infection rates lower than what would have been predicted during the baseline year.

CMS implemented financial reporting incentives in 2013 which greatly enhanced the number of reporting facilities [1]. From 2013 the MRSA SIR, using facility level data, improved or decreased from 0.96 to a nadir of 0.89 in 2014. It then increased to 0.99 in 2015, which approached its 2010–2011 baseline [2].

In 2015, the CDC re-normalized the SIR’s baseline to 1.0 [3]; for MRSA, this resulted in only minor changes for the value of the SIR, although the method of risk adjustment was also modified. Current risk adjustments are based on variables including the community prevalence of MRSA, medical school affiliation, facility type, average length of stay and number of ICU beds [4].

In the first quarter of 2020, acute care hospitals almost met the 2013 U.S. Dept. of Health and Human Services’ goal of a 25% reduction of invasive MRSA infections, but missed the revised 2020 goal of a 50% reduction in infections [5]. On July 12, 2022, the CDC announced a reversal in progress in the fight against antimicrobial resistance and at the same time MRSA was increasing in facilities, it was decreasing in the community [6].

The latest MRSA bloodstream infection data from the National Healthcare Safety Network (NHSN) (downloadable from https://data.cms.gov/provider-data/ ) has a data acquisition period of Oct. 1, 2020–Sept. 30, 2021. The data contained in this acquisition period had a facility level SIR for MRSA of 1.14, or worse than the 2015 baseline. Of note, only 35.7% of 4843 facilities had available data. Lastinger et al. observed that the national NHSN MRSA SIR fell to 0.77, a 23% reduction, in the first quarter of 2020, but then rose to 1.168 (preliminary data) in the first and third quarters of 2021 (See Fig. 1) [7]. Interestingly, the SIR fell in the second quarter of 2021, corresponding with a decrease in COVID-19 hospitalizations [7].

**Fig. 1:**
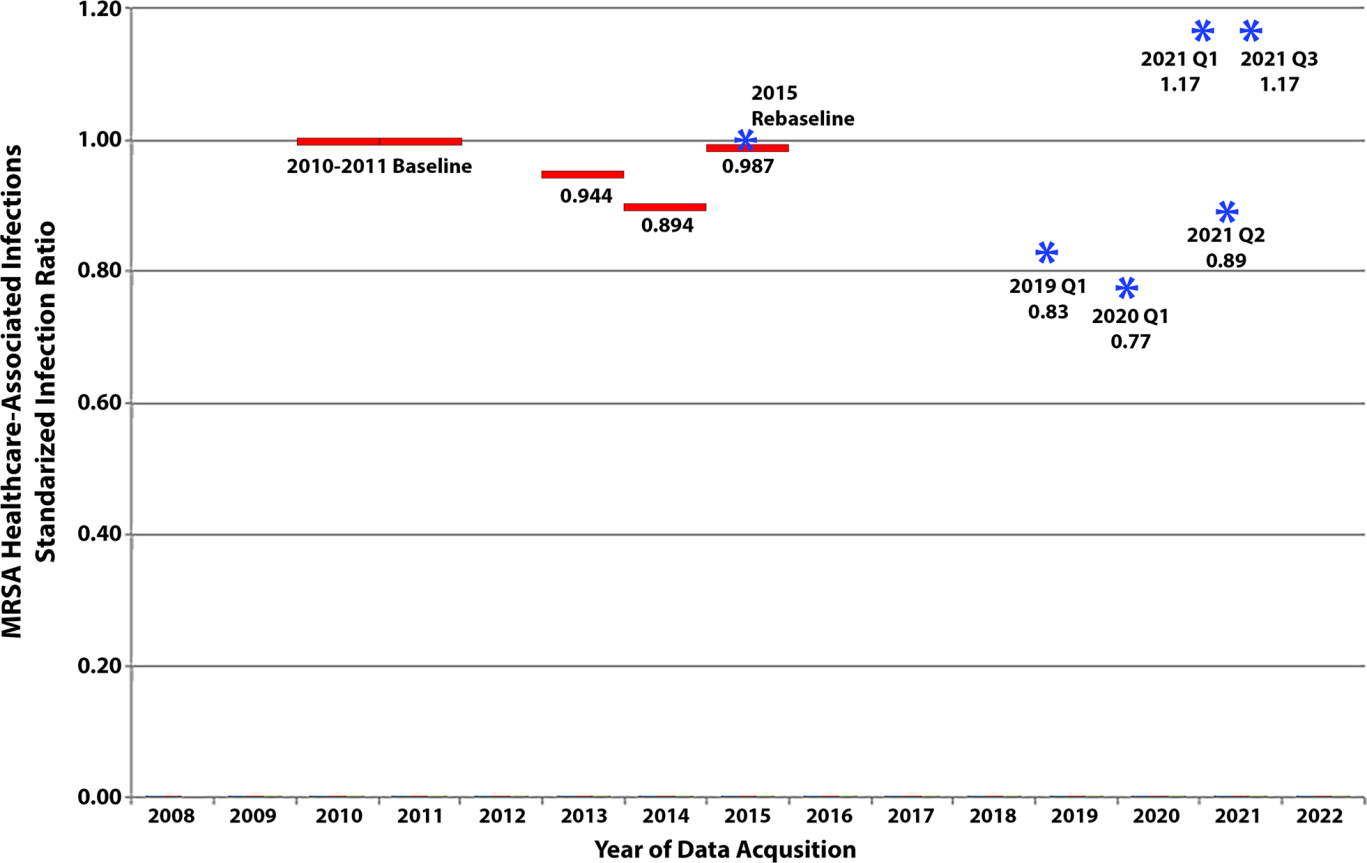
Standardized Infection Ratios for MRSA Healthcare-Associated Bloodstream Infections Reported to the NHSN. Facility Level Data, solid lines, from 2010 to 2015 was derived from Kavanagh et al. [2] National Level Data, asterisks, from 2015 to 2021 is available on the CDC Website at https://www.cdc.gov/hai/data/portal/covid-impact-hai.html

As the private sector has struggled to lower the rates of MRSA, the Department of Veterans Affairs has maintained a phenomenal reduction in MRSA Healthcare-Associated infections. Since Fiscal Year 2008, there has been an over 80% reduction at acute care facilities. The VHA maintained low rates of MRSA infections throughout the pandemic and obtained the lowest rates reported for Community-Living Centers and Spinal Cord Injury Units during the 2022 Fiscal Year (through the second quarter ending Mar. 31, 2022) with a 90.8% and 68.2% reduction from baseline, respectively [8]. During the Fiscal Year 2022, acute care facilities had a rate of MRSA infections, which approximated the lowest achieved rate in Fiscal Year 2019 (See Fig. 2). These rates have fallen over 50% since the 2011 study by Jain et al. [9], which reported that MRSA infections in Veterans Affairs’ acute care facilities through June of 2010 had decreased to 0.26 per 1000 patient days in non-ICU beds and 0.62 infections per 1000 patient days in ICU beds.

**Fig. 2:**
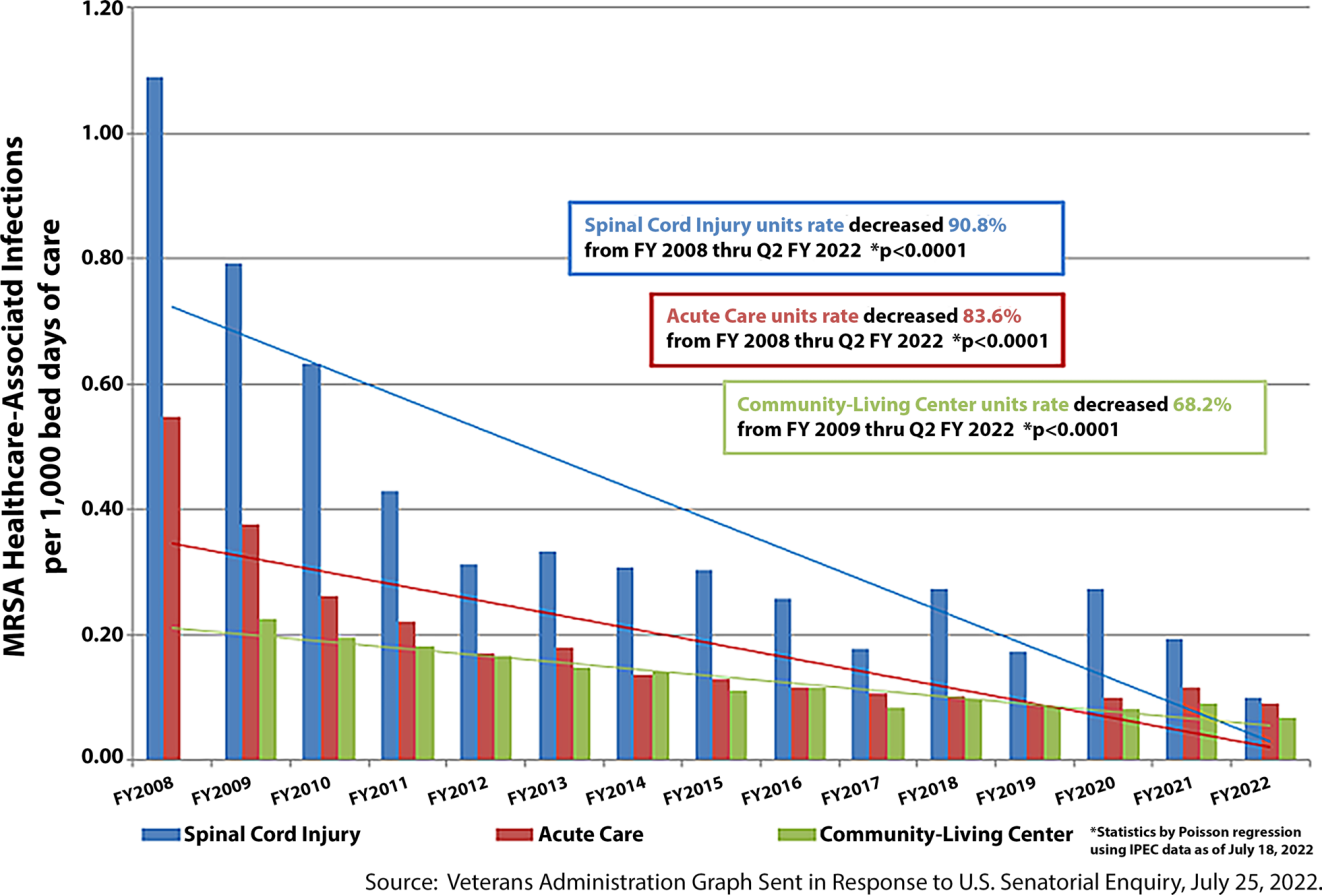
MRSA Healthcare-Associated Infection Rates-VHA Nationwide FY 2008 to FY 2022 (Q1–Q2)

The pandemic appeared to have placed substantial stress on the private sector with erasing gains in infection reduction. Specimens which are submitted to CDC’s Antimicrobial Resistance Laboratory Network, such as *C. auris* and gram-negative bacteria, decreased by 23% in 2020. In addition, a severe exacerbation of the nursing shortage took place in the private sector. As stated by Dr. Lisa Maragakis, Co-Chair of the CDC’s Healthcare Infection Control Practices Advisory Committee (HICPAC), during the March 24, 2022 meeting, “One of the main challenges I think that many of us are facing is an almost complete turnover in personnel on some of our units.“.

In contradistinction, the VHA by June of 2021 had reassigned over 3400 personnel to aid in staffing shortages in community nursing homes, private hospitals, Indian Health services and state veteran’s homes [10].

The VHA’s success in mitigating healthcare-associated MRSA infections can be attributed to not only the implementation of a uniform strategy of MRSA active surveillance and contact precautions for those colonized or infected at their facilities [8, 11], but also to their working environment and nursing resources. Despite the added demands of isolation protocols, the VHA was not only able to withstand the stresses of the pandemic but also aid other healthcare facilities in the community. These findings have implications not only regarding protocols for the control of MRSA but also for the need for transformation of our healthcare system in the private sector.

## Data Availability

NHSN data available from https://www.cdc.gov/hai/data/portal/covid-impact-hai.html and https://data.cms.gov/provider-data/.

## Abbreviations

CDC: Centers for disease control and prevention
FY: Fiscal year
MRSA: Methicillin-resistant *Staphylococcus aureus*
NHSN: National healthcare safety network
Q2: Second quarter
SIR: Standardized infection ratio
VHA: Veterans Health Administration
